# Minimal Setting Stroke Unit in a Sub-Saharan African Public Hospital

**DOI:** 10.3389/fneur.2019.00856

**Published:** 2019-08-07

**Authors:** Fode A. Cisse, Charlotte Damien, Aissatou K. Bah, M. L. Touré, M. Barry, A. B. Djibo Hamani, Michel Haba, Fode M. Soumah, Gilles Naeije

**Affiliations:** ^1^Department of Neurology, CHU Ignace Deen, Université Gamal Abdel Nasser Conakry (UGANC), Conakry, Guinea; ^2^Department of Neurology, CUB Hôpital Erasme, Université libre de Bruxelles (ULB), Brussels, Belgium

**Keywords:** stroke unit, stroke outcome, low to middle income countries, Sub-Saharan Africa, stroke mortality

## Abstract

**Introduction:** Sub-Saharan Africa (SSA) has the highest stroke prevalence along with a case fatality that amounts to 40%. We aimed to assess the effect of a minimal setting stroke unit in SSA Public hospital on stroke mortality and main medical complications.

**Materials and Methods:** The study was set in Conakry, Guinea, Ignace Deen public referral hospital. Clinical characteristics, hospital mortality and main medical stroke complications rates (pneumonia, urinary tract infections, sores and venous thromboembolism) of admitted stroke patients after the installation of a minimal stroke unit equipped with heart rate, blood pressure and blood oxygen saturation monitoring and portable oxygen concentrator (POST) were compared to a similar number of stroke patients admitted before the stroke unit creation (PRE).

**Results:** PRE (*n* = 318) and POST (*n* = 361) stroke, patients were comparable in term of age (61 ± 14 vs. 60 ± 14.8 years, *p* = 0.24), sex (56 vs. 50% males, *p* = 0.09), High blood pressure rate (76.7 vs. 79%, *p* = 0.44), stroke subtype (ischemic in 72 vs. 78% of cases, *p* = 0.05) and NIHSS (11 ± 4 vs. 11 ± 4, *p* = 0.85). Diabetes was more frequent in the PRE group (19 vs. 9%, p < 0.001). Mortality was significantly lower in the POST group (7.2 vs. 22.3%, *p* < 0.0001) as well as medical complications (4.1 vs. 27.7%, *p* < 0.001) and lower pneumonia rate (3.3 vs. 14.5%, *p* < 0.001).

**Conclusions:** Minimally equipped stroke units significantly reduce stroke mortality and main medical complications in SSA.

## Introduction

Stroke is a leading cause of death and disability worldwide (1). Good outcome in brain arteries occlusions is conditioned by early recanalization to prevent ischemic neuronal loss under the “time is brain” paradigm. To that aim, Healthcare policies in high income countries (HIC) underwent important structural modifications to allow as many patients as possible to benefit from recanalization therapy based on effective triage by advanced brain imaging and referral centers (2). This approach rely on both expansive imaging and endovascular intervention technique but has proved cost-effective to prevent death and disability from stroke in HIC (3). In recent years, the burden of stroke has moved from HIC to LMIC that now host 75 % of stroke mortality and 81 % of stroke-related disability (4). In low to middle income countries (LMIC), acute brain imaging facilities or stroke treatment referral centers are neither accessible nor affordable for the vast majority of the population (5) and most efforts to alleviate stroke burden are, therefore, based on primary prevention to control and limit risk factors (6, 7). Death and neurological deterioration after ischemic strokes are related to cerebrovascular complications due to lack of recanalization during the first hours (8) but can be attributed to medical complications in approximately 50 % of cases afterwards, mainly inhalation pneumonia, cardiac and thromboembolic diseases (9). These complications are partly prevented by acute surveillance in stroke units (SU). SU, in acute stroke management, consistently showed significant reduction of stroke burden and mortality, even in the earliest trials done when access to CT brain scanning was available for only a few patients which suggests that SU may contribute to stroke care in settings without easy access to brain imaging facilities (10).

In this work, we aim to assess the effect of a minimal setting SU on stroke mortality and medical complications in Conakry, Guinea, Ignace Deen public referral hospital that has no brain imaging facilities.

## Methods

### Local Setting

Guinea is a Sub-Saharan country of almost 12,000,000 inhabitants with an estimated medical doctor density of 7/100 000 inhabitants. Guinea is among the poorest countries in the world, ranking 171/192 based on the international money fund gross domestic product estimates. Healthcare system is pyramidal with the three national hospitals (Donka, Ignace Deen and Sino-Guinean) located in the capital, Conakry, of which only the Sino-Guinean is equipped with a computer tomography. According the World Health Organization data, two third of healthcare costs are supported by the patients and are therefore inaccessible to more than 40% of the population. In that context, cost-effectiveness has to be carefully balanced for patients to yield benefits from health intervention.

### Method

At Ignace Deen Hospital, a minimal setting stroke unit of three *acute* beds, separated from the other Neurology ward thirty beds, has been equipped in 2017 with heart rate, blood pressure and blood oxygen saturation monitoring and portable oxygen concentrator. There, patients are evaluated every 4 h, for clinical parameters, body temperature and National Institute of Health Stroke Score (NIHSS) by a dedicated stroke team that consists of five senior neurologists, eight neurologists in training, seven nurses, and three physiotherapists. Standard procedure, adapted from the American stroke association guidelines on *In-Hospital Management of acute stroke: General Supportive Care* (11) (For details, see **Addendum**), were implemented for fever, pneumonia and decubitus complication prevention. When patients are stabilized, they are transferred to *non-acute* beds in the neurology ward. Ignace Deen Neurology ward is keeping a stroke registry since 2015 of which clinical characteristics are recorded, based on the world health organization ‘'STEPS” (12) approach. Mortality at 28 days, Modified Rankin Scale (MRS) at 28 days when available and in-hospital pneumonia, urinary tract infections, sores and venous thromboembolism rates of admitted stroke patients after the installation of a minimal stroke unit during a 12 months period (January-December 2018, POST) were extracted and compared to stroke patients admitted before the stroke unit creation (January-December 2017, PRE). Patients included all had Brain CT during hospitalization that were realized at the medical imaging center, *Caisse Nationale de Sécurité Sociale*, a facility independent from Ignace Deen hospital located at 500 meters from the hospital.

Comparisons between the proportions were performed with respect to qualitative and quantitative variables. Proportions were compared between groups by Fisher exact test. Mann-Whitney test was performed to compare numeric variables in the two groups. Statistical significance was set after correction for multiple comparisons (13) using a Bonferroni correction at *p* < 0.004.

The local Ethics Committee of Ignace Deen Hospital (Comité National d'Ethique pour la Recherche en Santé, CNERS) approved the study, but waived the need for informed consent as only anonymous and operational monitoring data were collected and analyzed.

## Results: (Summarized in Table 1)

**Table 1:** Comparison of PRE and POST cohort clinical characteristics, outcome and complication.

	**PRE STROKE (*n* = 318)**	**POST STROKE (*n* = 361)**	***p*–value**
Age (years)	61 ± 14	60 ± 14,8	0.24
Males (%, *n*)	56 (178)	50 (178)	0.09
High Blood pressure (%, *n*)	76.7 (243)	79 (285)	0.44
Diabetes (%, *n*)	19.8 (63)	9.2 (33)	<0.0001
Ischemic stroke (%, *n*)	71.4 (227)	78 (281)	0.05
NIHSS	11± 4	11± 4	0.85
Death (%, *n*)	22.3 (71)	7.2 (26)	<0.0001
Stroke onset to admission (hours)	93 ± 70.6	92.3 ± 59.1	0.9
Complications (%, *n*)	27.7 (88)	4.2 (26)	<0.0001
Pulmonary infections	14.4 (46)	3.3 (12)	<0.0001
UTI	12.6 (40)	2.3 (10)	<0.0001
TE	0.9 (3)	0.3 (1)	0.88
Sores	6.6 (21)	0.8 (3)	<0.0001
MRS of stroke survivors	2.92 ± 0.94 (*n* = 227)	3.39 ± 1.05 (*n* = 249)	0.007

*NIHSS, national institute of health stroke scale; SU, stroke unit; UTI, urinary tract infection; TE, thrombo-embolic. MRS, modified rankin scale ranging from 0 (no symptoms) to 6 (death), >3: unable to walk unassisted*.

Three hundred and sixty-nine patients were included in the stroke registry after the installation of the SU (POST) and were compared to the three hundred and eighteen patients admitted for stroke when the SU did not exist (PRE). PRE and POST patients were comparable in term of age (61 ± 14 vs. 60 ± 14.8 years, *p* = 0.24), sex (56 vs. 50% males, *p* = 0.09), High blood pressure rates (76.7 vs. 79%, *p* = 0.44), stroke subtype (ischemic in 72 vs. 78% of cases, *p* = 0.05), NIHSS (11 ± 4 vs. 11 ± 4, *p* = 0.85) and time from stroke onset to SU admission (93 ± 70.6 vs. 92.3 ± 59.1 h, *p* = 0.9). Diabetes was more frequent in the PRE group (19 vs. 9%, *p* < 0.001).

Mortality was significantly lower in the POST group (7.2 vs. 22.3%, *p* < 0.0001) as well as medical complications (4.1 vs. 27.7%, *p* < 0.001) with lower pneumonia rate (3.3 vs. 14.5%, *p* < 0.001), urinary tract infections (UTI) (2.3 vs. 12.2%, *p* < 0.001) and sores (6.6 vs. 0.8%, *p* < 0.001). A trend for higher MRS in stroke survivors was found in the POST compared to the group (3.39 ± 1.05 vs. 2.92 ± 0.94, *p* = 0.007).

## Discussion

This study compared stroke mortality and main medical complications in a Sub-Saharan public hospital without brain imaging facilities, before and after the onset of a minimal setting stroke unit. In that context, surveillance of stroke patients in a minimal setting SU led to significantly lower mortality and main medical complications.

Our cohort matches the characteristics of prior reports in Sub-Saharan Africa in term of age, sex, high blood pressure and diabetes prevalence as well as in term of ischemic stroke proportion (13–15). The twenty-two percent mortality rate in Conakry stroke patients before the setting of the SU corresponds to the rates reported in the InterStroke study that pooled data from Mozambique, Nigeria, South Africa and Sudan and those from smaller series from Cameroon (13) and Congo (14) which suggests that our studied population is representative of Sub-Saharan LMIC stroke epidemiology. The stroke severity of our cohort, reflected by the NIHSS, is lower than the in high income countries stroke trials that validated thrombolysis where mean NIHSS was of 14 (16) and five points lower than the mean NIHSS retrieved from a meta-analysis of the mechanical thrombectomy trials (17). This relatively lower stroke severity can be explained by the fact that, in Guinea, most severe cases probably failed to reach the hospital due to the lack of hospital accessibility in terms of distance, cost and medical transportation means. Indeed, a survey realized at Ignace Deen Neurology ward in 2014 revealed that only 2% of stroke patients arrived in ambulance, 46 % came by public transportation, 27 % by personal car while the rest had to find other means. This lack of accessibility is also reflected by the time elapsed between the stroke onset and the admission in the SU closing to 4 days. These facts combined with the lack of money to sustain the high healthcare costs associated to severe diseases in Guinea suggest that an important part of the severe stroke cases remained and/or died at home or died underway. Paradoxically, the long time between stroke onset and SU admission is also likely to explain the efficiency of the SU on stroke mortality in Conakry. Indeed, stroke death in the first week is mostly accounted by acute cerebrovascular complications such as hemorrhagic conversion of the ischemic brain tissue or cerebral herniation due to edema of around the necrotic zone (8, 9). The patients hospitalized in Ignace Deen SU had for most of them, already survived the first days and were entering the period after stroke onset where medical complications are responsible for an important proportion of supplementary deaths. Pathological studies found that stroke deaths after the first week were, respectively, due to pulmonary embolism in 30%, inhalation bronchopneumonia in 27% and cardiac disease in 37% of autopsied cases (9). In our PRE group, this reality is well-reflected by the rates of main stroke medical complications such as bronchopneumonia, urinary tract infections and sores that are corresponds to the range of previous report in western countries, even in term of thromboembolic complications (18). However, while infections and sores are easily diagnosed and managed in medical contexts with few ancillary exams available, the 0.9% rate for thromboembolic complications probably underestimates the prevalence of such complication in LMIC settings, due to a diagnosis bias related to the lack of access to ventilation/perfusion scintigraphy or pulmonary arteries computed tomography angiography. Accordingly, the number of diagnosed thromboembolic complications was too low for statistical comparisons. Similarly, underlying cardiac diseases and complications could not be reliably recorded in our population for lack of technical means and are therefore missing from this report, an important caveat as deaths from cardiac affections accounts for a substantial proportion of delayed stroke deaths. The reported effect of the SU on stroke mortality in the Conakry context is likely to be related to the better prevention, detection and treatment of infectious and immobility complications at a time where medical complications are the main providers of increased mortality in stroke survivors. That fact also explains why SU care failed to improve the mean functional stroke disability outcome assessed by the MRS in POST compared to PRE: higher proportion of fragile and severely disabled patients were prevented to die from stroke complications by SU care, therefore increasing the mean MRS of stroke survivors in POST. This hypothesis is corroborated by the observed fall in bronchopneumonia, urinary tract infections and sores rates in the POST compared to the period prior to the SU onset. These results, obtained in a LMIC with no advanced brain imaging facilities accessibility, emphasizes the benefits of SU on death from stroke regardless of advanced brain imaging facilities accessibility and parallels findings obtained in HIC at a time when brain computed tomography was not yet a standard of care (19–21). In line with our study, in those seminal studies, one of the main effect of SU was to reduce the rate of stroke medical complications, mainly pneumonia, sores and venous thrombo-embolic diseases (19). Our report suggests that in contexts with low healthcare means and accessibility, SU could play a significant role in reducing stroke death and dependency. Especially in LMIC that now harbor the majority of the world stroke death and disability, public healthcare policies that enforce SU settings may prove highly effective in reducing disability-adjusted life years lost to stroke. SU spreading in LMIC may lead to more effective prevention of death and dependency from stroke than the development of single referral centers dedicated to recanalization therapy when patients struggle to pay the most basic healthcare interventions and arrive at the hospital several days after the stroke onset.

## Summary

Minimally equipped stroke units significantly reduce stroke mortality and main medical stroke complications in SSA and may constitute the base of stroke care regardless of advanced brain imaging accessibility.

## Addendum

Acute stroke care procedures:

– Supplemental oxygen
  ◦ In case of decreased level of consciousness
  ◦ To maintain blood oxygen saturation >94%
– Blood pressure control
  ◦ If blood pressure(BP) is above 220/120 in the first 24 h: BP is lowered by 15%.
  ◦ If BP is above 140/90 and the patient neurologically stable: antihypertensive therapy is started.
– Temperature
  ◦ Temperature above 38°C is treated by antipyretic and its cause looked for.
  ◦ Clinical pneumonia and urinary tract infections are treated by antibiotics when suspected.
– Glucose
  ◦ Blood Glucose levels are monitorized and insulin given to maintain the glycemia between 140 and 200 mg/dL.
– Dysphagia
  ◦ All stroke patients are clinically assessed: one tea spoon of water is given at the patient with his head in anteflexion. If coughing or alterations of mental state: no oral food for 24 h, then re-test: if coughing occurs, liquid and nutriments are given by nasogastric tube.
– Deep vein thrombosis and sore prophylaxis
  ◦ Regular patient mobilization and turning
  ◦ Regular skin assessment and maintenance of good skin hygiene.

## Data Availability

The datasets generated for this study are available on request to the corresponding author.

## Author Contributions

All authors listed have made a substantial, direct and intellectual contribution to the work, and approved it for publication.

### Conflict of Interest Statement

The authors declare that the research was conducted in the absence of any commercial or financial relationships that could be construed as a potential conflict of interest.
